# Fecal impaction: a systematic review of its medical complications

**DOI:** 10.1186/s12877-015-0162-5

**Published:** 2016-01-11

**Authors:** Blanca Serrano Falcón, Marta Barceló López, Beatriz Mateos Muñoz, Angel Álvarez Sánchez, Enrique Rey

**Affiliations:** Gastroenterology Department, Instituto de Investigacion Sanitaria del Hospital Clinico San Carlos (IdISSC), Madrid, Spain, 28040 Madrid, Spain; Department of Medicine, Universidad Complutense de Madrid, Spain, Plaza Ramón y Cajal, 28040 Madrid, Spain

**Keywords:** Fecaloma, Elderly, Complications, Classification

## Abstract

**Background:**

Fecal impaction (FI) is a common problem in the elderly and other at-risk groups, such as patients with a neuro-psychiatric disease. It has been associated with medical problems and high morbi-mortality. A systematic review of this topic might be useful to improve the knowledge in this area and helpful to make an appropriate and early diagnosis.

**Methods:**

A PubMed systematic search was performed using relevant keywords. Case reports published in English, Spanish or French till June 2014 were included if they had a diagnosis of FI and a medical complication secondary to it. Each case was classified based on its principal complication. The main objective is to create a classification of FI complications based on published clinical cases.

**Results:**

188 articles met inclusion criteria, comprising 280 clinical cases. Out of the total, 43,5 % were over 65 years old, 49 % suffered from chronic constipation, 29 % had an underlying neuropsychiatric disease and 15 % were hospitalised or institutionalised. A total of 346 medical complications secondary to FI were collected. They were divided according to gastrointestinal tract involvement and then classified based on their anatomical and pathophysiological mechanism into three groups: Complications secondary to fecaloma effect on the intestinal wall (73.4 %), on the intestinal lumen (14 %) and on adjacent structures (12.6 %).

**Conclusions:**

FI causes complications that might be fatal. The elderly, underlying neuropsychiatric disease and hospitalised or institutionalised patients integrate the high-risk group in which FI must be suspected. The first FI complications classification is presented to improve the knowledge about this entity.

## Keypoints

Fecal impaction, old people, neuro-psyquiatric disease, morbi-mortality, systematic review.

## Background

Faecal impaction (FI) is defined as a large mass of compacted faeces at any intestinal level that cannot be evacuated spontaneously [1]. It has been estimated that half of the institutionalised elderly suffer from it in the course of one year, and up to 7 % have impacted faeces if a rectal exams performed [2] The Elderly Represents The Main Risk Group For Presenting FI [3], although children and patients with underlying neuropsychiatric disease can also be involved in this problem [4] with significant consequences from a healthcare, socio-economic, and quality of life point of view.

From a pathophysiological perspective, faecal impaction causes an intraluminal pressure increase of the colon, and therefore ischemic phenomena are produced, which may lead to ulcer and colon perforation [5]. In addition, sustained dilation of the colon may cause megacolon and increased secretion at this level, which, combined with the decreased sphincter tone in the elderly, gives rise to anal incontinence and diarrhoea in this patient group [6]. Faecal impaction may also cause mechanical obstruction of the colon [7] and compress nerve, vascular, or solid organ structures by a mass effect.

Faecal impaction is also responsible for hospital admissions [8] and increased morbidity-mortality [9]. In spite of this and the number of clinical cases published on the matter since 1894 [10], the complications secondary to FI have scarcely been investigated. Only reviews of stercoraceous perforation, one of the most commonly described complications, have been published [11].

The objective of this qualitative- systematic review is to collect the published cases of complications of FI, evaluate them, and create a classification based on said analysis. Secondary objectives are to explore whether the elderly are especially vulnerable to a higher risk of morbidity-mortality resulting from FI, and whether there exists a clinically identifiable at-risk profile. This may allow for greater knowledge of faecal impaction and its complications.

## Methods

### Systematic review

The PubMed database was the source used to perform the systematic review. The search was limited to case reports published in English, Spanish, or French, up to June 31, 2014.

The search strategy used the combination of “faecal impaction” with the words “complications”, “obstruction”, “incontinence”, “stercoral ulcer”, “stercoral colitis”, “stercoral perforation”, “fistula”, “dystocia”, “bleeding”, “perforation”, “megacolon and megarectum” and “dystocia”, resulting in a total of eleven combinations, connected by the preposition “and”. Separately, the search was expanded with the terms “stercoral ulcer”, “stercoraceous ulcer”, “stercoral perforation” and “stercoraceous perforation”. Finally, we ensured the inclusion of the bibliographical references from the four previously published reviews on stercoraceous perforation (Serpell et al, Guyton et al, Maurer, Chakravartty et al). The summaries and clinical descriptions were reviewed by two investigators.

Clinical cases diagnosed of FI and any complication secondary to it were included. **Fecal impaction** was defined as a large mass of compacted faeces at any intestinal level that cannot be evacuated spontaneously [1]. Cases not meeting the previously mentioned criteria were excluded, as were barium or bezoar impaction, acute appendicitis secondary to fecalith, spontaneous intestinal perforation in default of FI and open clinical cases. The articles whose full text was not accessible electronically or through the National Interlibrary Network were not included in the review.

### Data collection

Data included case’s FI medical complications, age (categorised into children up to 15 years old, adults, and persons over 65 years old), gender, place of residence (institutionalised or not), medical histories (classified into neuropsychiatric disease, digestive issues (scleroderma, Chagas Disease, prior intestinal surgeries, and anorectal malformations), heart disease, chronic kidney failure, chronic constipation, consumption of laxatives and habitual drugs) and the need for emergency surgery and prognosis .

Article’s characteristics, including the bibliographical citation, number of clinical cases included in each one, and the previously described data were collected in *Microsoft Excel 2010*. The descriptive analysis was performed with said program. All of the categorical variables were added up and expressed in a percentage.

## Results

**Fig. 1:**
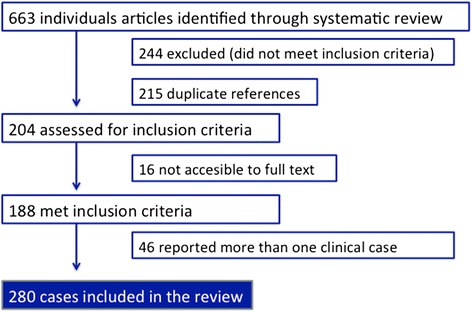
Study selection process. Breakdown of study selection process

The initial search yielded 663 references and 204 were considered for the review (244 did not meet inclusion criteria after abstract assessment and 215 were duplicate references). A total of 188 met inclusion criteria after checking the full text, comprising 280 cases regarding complications of FI. Breakdown of study selection process is given in Fig. 1.

### Description of the published cases

#### Fecal impaction complications: risk factors

Half of published cases were between 15 and 65 years old (139 cases) 122 cases were over 65 years old, and only 19 cases, were under 15 years old. The majority were women (163 cases, 58 %) with the highest proportion of them in the elderly group (67 %).

Chronic constipation (CC) was present in 50 % of the cases, 19 % were not constipated prior to the faecal impaction episode, and there was no complaint of CC in the 31 % of the remaining cases. Out of the 139 suffering chronic constipation only 34 stated habitual laxative consumption. It is more common in women, especially in older women (68 % of cases over 65 years old with chronic constipation were women).

Neuropsychiatric disease (76 cases, 27 %) stands out as a significant at-risk group for the development of FI in all age groups. Gastrointestinal tract damage from scleroderma, Chagas Disease, prior intestinal surgeries, and anorectal malformations were described in 31 cases. Chronic kidney failure is described in 28 cases. Regarding to residence, 14 % of the cases were hospitalised or institutionalised when FI complication was diagnosed.

#### Other collected data

Abdominal pain was the most common symptom every age groups (43 %), followed by constipation (18 %), nausea and vomiting (15 %), and abdominal distension (9 %). Other less common symptoms were diarrhoea, faecal incontinence, or urinary symptoms. Sigmoid colon was the most common location of FI (68 %), followed by rectum (51 %). Other locations have been described less often (28 %). Treatment was surgical in 198 cases, out of which 190 were performed urgently (68 % del total). FI and its complication were resolved conservatively, including manual disimpaction, in 22 % of the total. The remaining 7 % of the cases were diagnosed by means of an autopsy.

#### Prognosis

Death secondary to FI complications was reported in 78 cases (29 % of the total). Prognosis was favourable in 184 cases and unknown in 18. (Table 1). Death was more frequent in the group aged over 65 years (32 % out of published deaths) compared to 28 % in the group of adults from 15 to 65 years old. Respect to comorbidity, death was more frequent in patients with neuropsychiatric disease (30 %) and chronic renal failure (43 %). 54 % of patients with prior hospitalisation had a fatal prognosis secondary to faecal impaction complication.

**Table 1:** Prognosis according to age group, history and classification groups for faecal impaction

Prognosis	N Death	N Favourable	N Unknown	N total
Total (N cases)	78 (28 %)	184 (66 %)	18 (6 %)	280
Age range (years)				
>15	0	18 (95 %)	1 (5 %)	19
15–65	39 (28 %)	90 (65 %)	9 (7 %)	138
≥65	39 (32 %)	76 (62 %)	8 (6 %)	123
History				
Neuropsiquiatric disease	23 (30 %)	49(65 %)	4 (5 %)	76
Chronic renal failure	12 (43 %)	14 (50 %)	2 (7 %)	28
Prior hospitalisation	22 (54 %)	18 (44 %)	1 (2 %)	41
Classification group				
Intestinal wall	72 (31 %)	141 (61 %)	19 (8 %)	232
Intestinal lumen	13 (30 %)	30 (68 %)	1 (2 %)	44
Adjacent structures	5 (12,5 %)	33 (82,5 %)	2 (5 %)	40

### Published fecal impaction complications

A total of 346 complications secondary to FI were described among the 280 included cases. The majority of them, 224, presented only one complication, and in 46 and 10 cases 2 and 3 complications were described, respectively.

Collected complications were classified according to gastrointestinal tract involvement (Table 2).

**Table 2:** Schematic display of FI complications distribution

FI complications with gastrointestinal tract damage	No. of cases	FI complications without gastrointestinal tract damage	No. of cases
Intestinal perforation	145	Obstructive uropathy	27
Intestinal obstruction	35	Dystocia of the birth canal	5
Stercoraceous ulcer	30	Urinary bladder damage	4
Intestinal pseudo-obstruction	9	Compression of vascular and nerve structures	4
Stercoraceous colitis	8	Septic shock	6
Fistulas (rectovaginal, sigmoid uterine and anorectal)	8	Respiratory distress	3
Fecal incontinence	4	Acute pulmonary oedema	2
Paradoxical diarrhoea	3	Systemic inflammatory response syndrome	2
Pericolic abscesses	2	Pneumothorax	1
Colostomy prolapse	1	Gluteal abscess	1
llcocaccal invagination	2	Interstitial nephritis	1
Complex inguinal hernia	1	Acute pyelonephritis	1
Diverticulitis	1	Chorioamnionitis	1
		Premature delivery with foetal death	1
		Hepatic encephalopathy	1
		Hypotrophy of liver and spleen	1
		Obstruction of dialysis catheter by fecaloma	1

### Faecal impaction complications: classification

The collected complications from published cases were organised into three principal groups according to their pathophysiological and anatomic mechanism: fecaloma damage on the wall, on the intestinal lumen or on adjacent structures.

A total of 316 complications were included in this classification out of the 346 collected in the review. “Faecal impaction effect on intestinal wall” was the most numerous group, followed by “Faecal impaction effect on the intestinal lumen” and by “Faecal impaction and adjacent structures”, as displayed in Table 3. The mixed group “Other complications” comprises the 30 remaining complications not included in the mentioned classification, making up 9 % of the total (Table 4).

**Table 3:** Schematic display of the distribution of FI complications included in the classification

Intestinal wall	232	73.4 %
Increased secretion	7	3 %
Diarrhea	3	1.3 %
Incontinence	4	1.7 %
Increased distensibility: megacolon	34	14.6 %
Increased pressure	191	82.3 %
Intestinal perforation	145	62.5 %
Stercoraceous ulcer	30	13 %
Stercoraceous colitis	8	3.4 %
Fistula	8	3.4 %
Intestinal lumen	44	14 %
Obstruction	35	11 %
Partial obstruction	9	3 %
Extracolon damage	40	12.6 %
Obstructive uropathy	27	8.5 %
Bladder	4	1.26 %
Gynaecological: dystocia	5	1.6 %
Vascular or nerve structures	4	1.26 %

**Table 4:** Schematic display of the distribution of the total complications of faecal impaction, including the “Other” group

Intestinal wall	232	67 %
Intestinal lumen	44	13 %
Adjacent structures	40	12 %
Other	30	9 %
TOTAL COMPLICATIONS	346	100 %

Cases of females aged between 15 and 65 years old predominated in the three classification groups. In terms of medical history, chronic constipation was present in 56 % of the patients that suffered complication from fecaloma on the intestinal wall, and in 44 % and 28 % of the cases of complication along adjacent structures and the intestinal lumen, respectively. 52 % of complications along adjacent structures were in patients with underlying neuropsychiatric disease. It was slightly lower in complications involving the wall (25 %) or intestinal lumen (21 %). As regards the need for emergency surgery, it was more common in cases of fecaloma damage on the intestinal wall (79 %), and on the intestinal lumen (57 %). Medical treatment was the most common in the group with fecaloma damage on adjacent structures (81 %).

Death was more frequent along the published cases with complications resulting from the effect of fecaloma on the intestinal wall (32 %), followed by, cases with fecaloma damage on the intestinal lumen (28 %) cases with damage to adjacent structures (13 %) (Table 5).

**Table 5:** Table of results in the three classification groups for the complications of faecal impaction

		Intestinal wall	Intestinal lumen	Adjacent structures
Age	<= 15	3 %	4 %	19 %
	(15. 65]	50 %	62 %	39 %
	>65	47 %	34 %	42 %
Sex	Male	41 %	43 %	42 %
	Female	59 %	57 %	58 %
History	Neuro/psychiatric	25 %	21 %	50 %
	Prior hospitalisation	15 %	23 %	8 %
	Chronic constipation	56 %	28 %	44 %
Reason for visit	Abdominal pain	67 %	77 %	36 %
	Chronic constipation	27 %	45 %	14 %
	Abdominal distension	13 %	28 %	11 %
	Diarrhoea	1 %	11 %	3 %
Treatment	Emergency surgery	79 %	57 %	19 %
Prognosis	Deaths	32 %	28 %	14 %

## Discussion

**Fig. 2:**
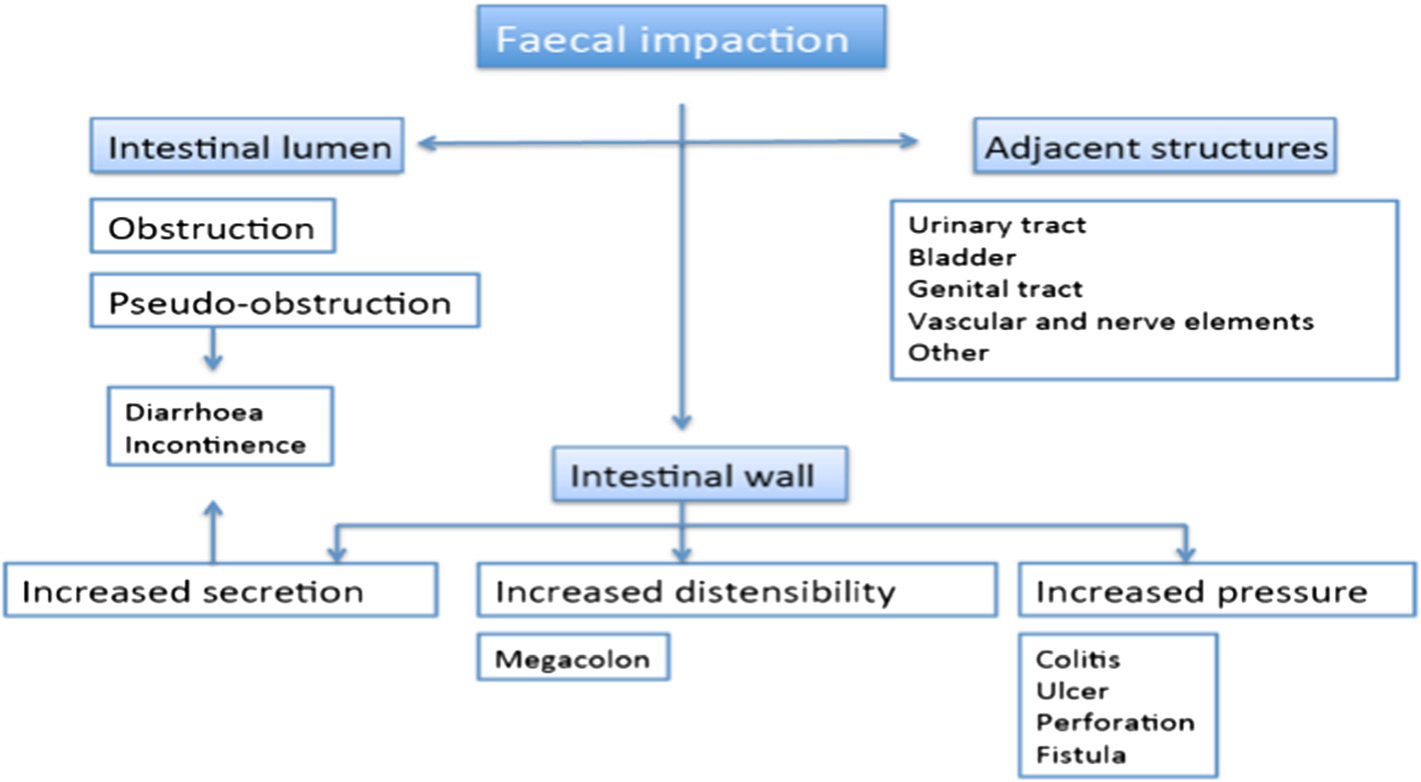
Classification of fecal impaction complications. Schematic display of the classification of faecal impaction complications

Our study reports the first classification about fecal impaction complications. After listing the total of complications and classifying them according to gastrointestinal tract damage, it seemed reasonable to organise them based on their pathophysiological and anatomical mechanism. They were classified into three principal groups: complications resulting from the principal effect of the fecaloma on the wall, the intestinal lumen or the adjacent structures, as shown in the display in Fig. 2.

### Fecaloma and intestinal wall

#### Increased pressure

Fecalomacontact on the intestinal wall has a pressure effect. The increase of intraluminal pressure over the capillary perfusion one causes ischemic phenomena that give rise to a local inflammatory reaction and necrosis [5] leading to ulcers and a possible subsequent perforation [12, 13]. These processes are called colitis, ulcer and stercoraceous perforation, whose diagnostic criteria are well established. Stercoraceous perforation is defined as a perforation secondary to a mass of faeces, with evidence of pressure ulcer and acute inflammatory reaction around it, in the absence of another cause of the perforation [14].

This type of lesion most often occurs in the sigmoid and rectosigmoid colon, due to the harder consistency of the faeces, smaller diameter of the colon and poorer vascularisation especially on the anti-mesenteric border [15].

Cases with demonstrated colitis on the tissue sample or wall thickening by abdominal ultrasound, without evidence of ulcer or perforation, were classified as “colitis”. We consider it to be aprior pathophysiological stage to the appearance of more serious complications such as ulcer or intestinal perforation. Following this approach, cases without perforation were classified as ulcer. The cases of ulcer or perforation with no clear evidence of faecal impaction were excluded from this review.

Stercoraceous perforation is not a very common cause of perforation in clinical practice [16]. However, this constitutes the group with the highest number of cases in the review, probably due to a publication bias.

### Increased secretion

Fecal impaction may cause rectal distension. The continuous contact between faeces and wall may cause mucous membrane irritation and a resulting increase in mucous secretion. Furthermore, FI may cause prolonged relaxation of the internal anal sphincter, a hypothesis proven in children [17] though not in adults [18]. It appears more accepted that an increased of secretion at that level, combined with the decreased tone of the sphincters in the elderly and the neuropathy component of the pudendal nerve [19] explains the anal incontinence and diarrhea (paradoxical) in this patient group [6]. Moreover, manual disimpaction manoeuvres practised as a remedy for faecal impaction may contribute to the damage to the internal anal sphincter.

Anal incontinence is the most common complication of FI, and causes a significant decrease in the quality of life [9]. Its prevalence is up to 8.3 % in non-institutionalised adults [20] and increases up to 50 % in the institutionalised elderly [21, 22]. Nevertheless, only 4 cases with this complication were included in this review, probably due to a publication bias.

### Increased distensibility

The disruption of colonic transit caused by faecal impaction may cause sustained increases in wall distensibility and therefore dilation of the rectum or colon by a mechanism scarcely investigated. Entities presenting colon dilatation, such as Hirschsprung’s Disease, Chronic intestinal pseudo-obstruction, or Chagas Disease may easily coexist with FI, while not necessarily being the cause of the diseasebut rather a consequence [23]. It is difficult to assess whether recurring FI contributes to megacolon/megarectum, or whether idiopathic megacolon leads to the development of FI by means of a disturbance in colonic motility. Colonic volvulus must be discarded as a complication of megacolon in these patients [24]. Since chronic constipation and megacolon are considered risk factors for colonic volvulus [25], faecal impaction could be involved in the pathogenesis; however, future researches should be done to clarify it [26].

On the other hand, It is mandatory to distinguish toxic megacolon, reported in three cases included in this review, characterised by colonic distension of more than 6 cm in the presence of colitis, and signs of systemic toxicity, because of its high related mortality [27]. It is more common in ulcerative and pseudomembranous colitis [28], but may be secondary to FI [29] caused by an increased of intraluminal pressure, according to the previously described mechanism.

### Faecaloma and intestinal lumen

FI is a rare cause of colon obstruction [30]. However, this represents the second group in terms of frequency among the reviewed complications. From a pathophysiological point of view, lumen obstruction causes colonic pressure increase with the aforementioned complications development. Prompt identification may avoid ischemic phenomena, perforation, and increased secretion, which would cause the so-called “paradoxical or overflow diarrhoea”. Partial obstruction is considered if obstruction is not total and must not be confused with the term Intestinal pseudo-obstruction, in which colon dilation is idiopathic [31]. Intestinal obstruction contraindicates treatment with oral solution [32], and its early identification therefore changes the patient’s treatment and prognosis. Differential diagnosis must be done with colonic volvulus, which represents he third cause of colonic obstruction, typical in chronic constipation and elderly people [33]; clinical manifestations present with the triad of constipation, progressive abdominal distension, and severe abdominal pain.

### Faecaloma and adjacent structures

The impacted faeces mass effect may cause compression on adjacent structures. The link between faecal impaction and urinary system has been the most widely investigated one so far. Highly distended rectum pressure on the bladder or the urethra has been the main reported mechanism to explain urinary retention in patients with FI [34, 35]. Urinary incontinence, more common than retention in geriatric age [36], may be due to urgency, overflow, hyperactivity of the detrusor [37] or to bladder irritation. FI- dystocia association has been mainly reported in patients with underlying psychiatric disease [38]. It also appears to be secondary to FI mass effect, although the mechanism in this group has not been investigated due to its low frequency.

### Faecal impaction and risk groups

The elderly and neuropsychiatric patients remain the main principal risk groups for faecal impaction development [3, 9, 39]. However, the adult age group (15–65 years old) has been the most frequently reported,. It may be explained by a publication bias, most exceptional cases are reported instead of more frequent ones in clinical practise. Neuropsychiatric disease has been the most frequently published comorbidity and chronic constipation was more frequent among this group. Colonic motility and rectal sensitivity impair, combined with the lack of mobility may be factors that contribute to its development [40]. Anticholinergic and antiserotoninergic drugs effects could also be a relevant factor [41]. Patients, principal caretakers and healthcare workers lack of knowledge may encourage the perpetuation of the problem and the development of FI complications.

Complications do not follow a specific or predictable pattern when are more than one in a same case, although available data have been taken from published cases and conclusions should not be drawn in this regard.

### Outcome

A total of 78 deaths were reported in the reviewed cases. It was more common among the group of “fecaloma effect on the intestinal wall”, since this group’s main complication is colon perforation, with a high associated mortality rate. Mortality rate previously reported in stercoraceous perforation case series was similar: 33 % [10]. Death was more frequent in the group aged over 65 years (32 % out of published deaths) compared to 28 % in the group of adults from 15 to 65 years old. Respect to comorbidity, death was more frequent in patients with neuropsychiatric disease and chronic renal failure, probably due to the associated comorbidities in these patients. These results confirm that risk groups, previously mentioned, are also more likely to have a fatal prognosis. Special attention must be done in this regard.

### Faecal impaction: how to avoid a complication

Investigate faecal impaction and a possible complication in certain risk groups of patients is mandatory when a sign or a symptom is presented. A complete history and physical examination must be performed, including a digital rectal examination, but it does not exclude a faecal impaction in more proximal levels. A radiologic imaging, such as an acute abdominal series or computed tomography (CT) will lead to a prompt identification and directed treatment, avoiding more severe complications [32]. Treatment options include manual desimpaction when the faecal mass is palpable in the rectum. Softening of hardened stool and stimulation of evacuation with enemas or suppositories is often helpful, as well as oral lavage with polyethylene glycol solutions for proximal faecal masses [39]. Surgical evaluation must be done when peritonism signs are present [12]. However, prevention with laxatives and fiber and water intake must be considered as it is a recurrent problem [3].

### Limitations

Since this is a systematic review, results concern published cases, and are therefore not applicable to routine clinical practice, although it leads to a better knowledge of possible complications of this entity.

Search criteria used in this review may fail to include some papers about faecal impaction complications, since only one bibliographic database has been evaluated, language has been an exclusion criteria, and some special cases have not been included. Furthermore, the articles whose full text was not accessible were not included in the review.

Some of the complications may be classified into more than one group, such as faecal incontinence, which could cause classification biases.

This classification is proposed in based to the results of a descriptive study and a anatomical and pathophysiological mechanism, not based on statistical modelling. This fact makes the classification could be further refined or improved.

In this regard, Future researches may be done, applying this knowledge in clinical practice to obtain real results about an understood problem like faecal impaction and its complications.

## Conclusions

The first systematic review on faecal impaction complications is presented. It allows to list every reported complications and create a classification based on anatomical and pathophysiological concepts: Fecaloma damage on the wall, the intestinal lumen or adjacent structures. This shows that such a common pathology in routine clinical practice can become complicated, and it explains how it may occur. It may be helpful to early identify FI and a probable complication in the main risk groups: the elderly, institutionalised, and patients with neuropsychiatric disease. In this way, a fatal outcome may be avoided.
